# Rhino breaks the deadlock in *Drosophila* testis

**DOI:** 10.1371/journal.pgen.1009702

**Published:** 2021-09-02

**Authors:** Anahi Molla Herman, Emilie Brasset

**Affiliations:** 1 Collège de France, CIRB, CNRS INSERM UMR 7241, PSL Research University, Paris, France; 2 iGReD, Université Clermont Auvergne, CNRS, INSERM, Faculté de Médecine, Clermont-Ferrand, France; College de France CNRS, FRANCE

In the early 2000s, Aravin and colleagues discovered, in the *Drosophila melanogaster* testis, a new class of small regulatory RNAs initially named repeat-associated small interfering RNA (rasiRNAs). rasiRNAs were first described as regulating a protein-coding gene. They are derived from the *Suppressor of Stellate [Su(Ste)]* locus located on the Y chromosome and were shown to target the X-linked *Stellate* repeated genes by sequence complementarity. *Stellate* genes encode proteins with homology to the regulatory β subunit of the protein kinase CK2. *Stellate* repression occurs during male gametogenesis and is essential for male fertility. These small RNAs were renamed as PIWI-interacting RNAs (piRNAs); they are 23 to 29 nucleotides (nt) long and bind to proteins of the PIWI family. Accordingly, piRNA pathway mutants are sterile, and they contain crystalline aggregates of *Stellate*-coded protein [1].

**Fig 1:**
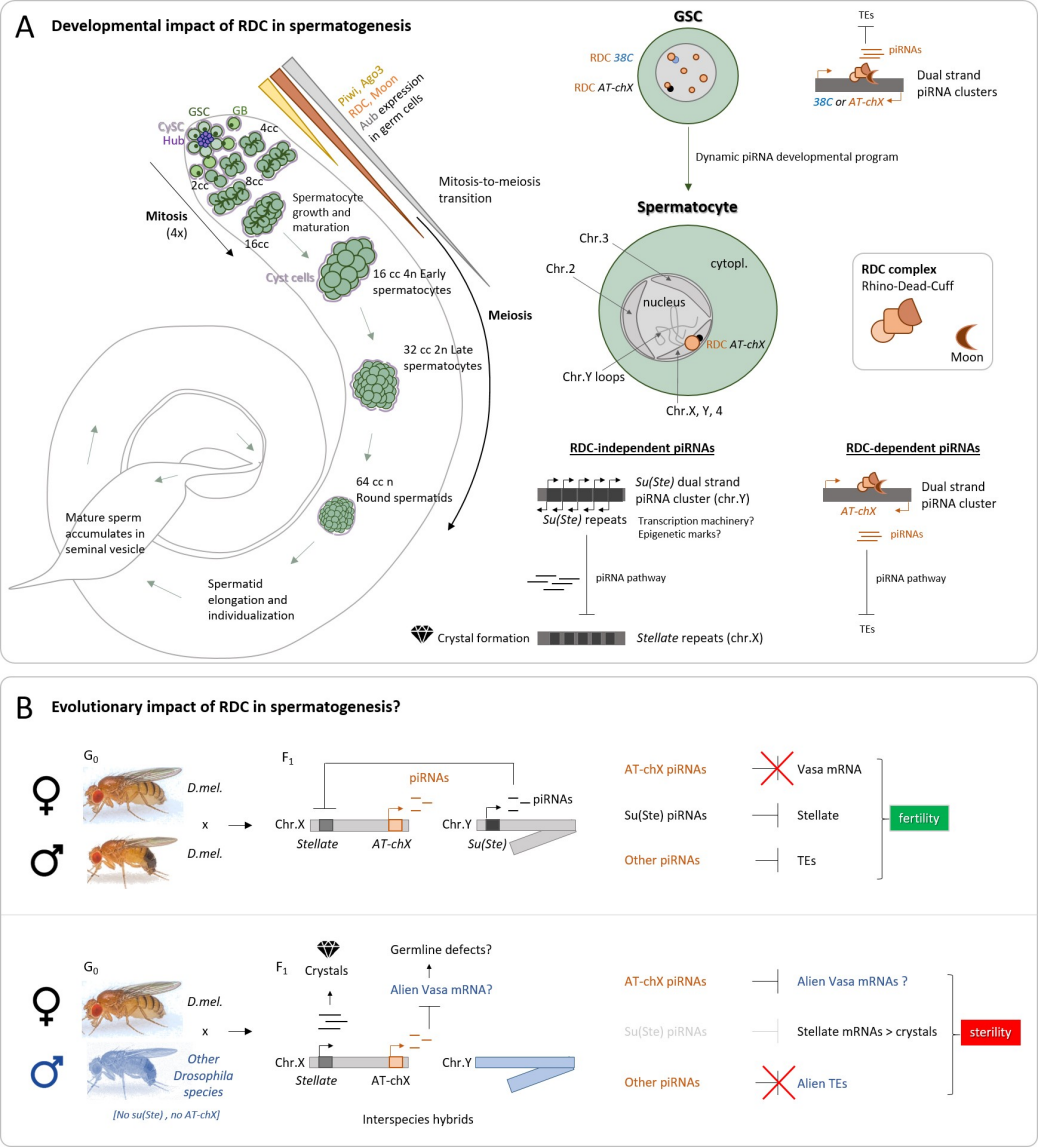
**(A)** Developmental impact of RDC in spermatogenesis. Left: The *Drosophila melanogaster* testis is depicted with the germline in green and the somatic cells in violet (CySCs). In the mitotic region, GSCs divide to form GBs and cysts of 2-, 4-, 8-, or 16-cell spermatogonia. Then, spermatogonia grow and mature to form spermatocytes that enter meiosis. After spermatid elongation and individualization, sperm will accumulate in the seminal vesicle. RDC, PIWI, Ago3, and Aub levels of expression are depicted in orange, yellow, and gray. Right: GSC and spermatocyte magnifications. In GSC, Rhino protein is localized in several foci, decorating the *38C* and *AT-chX* piRNA dual-strand clusters, among others. In spermatocytes, *38C* is not expressed anymore, and *AT-chX* loci is highly expressed and colocalizes with a single RDC foci. *Su(Ste)* dual-strand transcription is RDC independent. *Su(Ste)* piRNAs together with piRNA pathway proteins silence *Stellate* genes to avoid crystal formation. **(B)** Evolutionary impact of RDC in spermatogenesis. Top: *D*. *melanogaster* female and male are depicted. *Su(Ste)* piRNAs produced from the Y chromosome avoid *Stellate* expression and crystal formation. *AT-chX* piRNAs do not have enough homology with vasa to silence it in *D*. *melanogaster*. Bottom: A cross of a *D*. *melanogaster* female with another species produces interspecies sterile hybrids, since *Stellate* genes form crystalline aggregates in their spermatocytes when expressed, and AT-chX could silence “alien vasa,” altering the germline. cc, cell cyst; CySC, cyst stem cell; GB, gonioblast; GSC, germline stem cell; piRNA, PIWI-interacting RNA; RDC, Rhino–Deadlock–Cutoff; TE, transposable element.

piRNAs were then discovered in mammals and in most other animal germ cells [2]. Nowadays, we know that piRNAs are mainly devoted to protecting the genome from active mobile genetic elements in the metazoan germline. In *D*. *melanogaster*, both sexes depend on a functional piRNA pathway for their fertility. Our understanding of piRNA biogenesis and function comes predominantly from studies of the female *Drosophila* germline, but the male’s piRNA pathway remains poorly understood. Previously, studies reported that many proteins involved in the female piRNA pathway are also required for male fertility and *Stellate* silencing in the testis, supporting the conservation of piRNA pathway machinery in both sexes [3]. While Aub is expressed broadly from germline stem cells (GSCs) to primary spermatocytes, Ago3 and PIWI were detected only in mitotically dividing germline cells (GSCs and spermatogonia), indicating stage-specific modulations of the piRNA pathway [4–6] (Fig 1A). The 2 most active piRNA clusters in the testis are dual strand: *Su(Ste)* genes and *AT-chX* [5,7,8]. Interestingly, Aravin’s team have recently defined novel piRNA clusters in *D*. *melanogaster* spermatogenesis and show piRNAs adaptation, dependent on sex-specific expression of transposons [8]. Dual-strand piRNA clusters generally lack promoters, and their expression depends on the Rhino–Deadlock–Cutoff (RDC) complex that licenses noncanonical transcription. The RDC is anchored to H3K9me3-marked chromatin via Rhino’s chromodomain. This process involves 5′-end protection of nascent RNAs and suppression of transcription termination [9]. Importantly, since fertility in male *Rhino* mutants was not compromised and stellate crystals were absent, RDC function in spermatogenesis was neglected [10].

In this issue of *PLOS Genetics*, Chen and colleagues explored whether piRNA loci in the *D*. *melanogaster* testis use the same RDC noncanonical transcription mechanism as in the female germline [11]. Unexpectedly, a detailed examination of male fertility by sperm exhaustion assays revealed germline defects and subfertility in RDC mutant males. A careful spatiotemporal analysis showed that the RDC complex is not ovary specific. RDC assembled also in early spermatogenesis to regulate piRNA cluster expression required for an efficient transposable element (TE) silencing in the testis. Like in ovaries, they observed that RDC marks all dual-strand piRNA clusters, except *Su(Ste)*. Furthermore, Moonshiner (a paralog of transcription initiation factor TFIIA-L) then links the RDC to RNA polymerase II to initiate a promoterless noncanonical transcription.

One striking result from Chen and colleagues is that transcription of the *Su(Ste)* dual-strand cluster is Rhino independent, and *Stellate* genes are properly silenced in *Rhino* mutants [11]. Similarly to piRNAs found in some arthropods and mammals, the *Su(Ste)* locus may be canonically transcribed by RNA polymerase II, with initiation occurring from convergent promoters. Sense transcripts of *Su(Ste)* are spliced and polyadenylated [12]. This is consistent with the *Su(Ste)* locus lacking RDC, which would actually suppress splicing and polyadenylation if present. Importantly, when *Su(Ste)* repeats start to be expressed in spermatocytes during the mitosis-to-meiosis transition, the general transcription machinery is replaced by testis-specific machinery, like testis-specific TBP-associated factor (tTAF), testis-specific meiotic arrest complex (tMAC), and the coactivator mediator [13]. It remains to be addressed whether this testis-specific machinery is required for *Su(Ste)* expression.

In this new study, Chen and colleagues showed that RDC first forms multiple nuclear foci in GSCs and spermatogonia due to its association with multiple dual-strand piRNA clusters and then concentrates in spermatocytes in a single dot corresponding to a unique piRNA cluster, the X-linked *AT-chX* locus [11]. Importantly, the spermatocyte stage is known to have some unique features. (1) Chromosomes get organized in 3 distinct chromosome territories (2; 3; X, Y, 4) [14]. (2) The X chromosome is transcriptionally down-regulated, while the heterochromatin-rich and gene-poor Y chromosome is highly expressed due to Y-loop formation [15,16]. (3) Pericentromeric satellite sequences within the X, Y, and fourth chromosomes get together and are decorated with D1 (a multi-AT-hook satellite DNA-binding protein involved in chromocenter formation) [17]. Thus, 2 intriguing key questions arise from this study: (i) Why does the X-linked pericentromeric *AT-chX* locus not colocalize with pericentromeric D1? (ii) How does *AT-chX* become highly expressed at this stage? One possibility might be that *AT-chX* loops out to be excluded from the down-regulated X chromosome, similarly to the Y chromosome. Also, we cannot exclude that the single Rhino foci results from the concentration of multiple piRNA clusters, similarly to rDNA that cluster in the nucleolus to be transcribed by RNA-pol-I in many organisms [18]. Future single-cell transcriptomics and high-throughput chromosome conformation capture (HiC) experiments performed in the testis should help to better understand *AT-chX* and other piRNA cluster expression and nuclear organization during spermatogenesis.

Finally, even if *AT-chX* is the second most expressed piRNA cluster in the testis after *Su(Ste)*, its function still remains poorly understood. One possibility is that *AT-chX* could be a master locus required for the piNG-body formation, as *flamenco* is necessary for the Yb body formation in ovarian somatic cells [19]. Indeed, the piNG-body emerges at the spermatocyte stage and contains proteins similar to the female nuage [20]. It has also been proposed that *AT-ChX* piRNAs could participate together with the *Su(Ste)* system to assist the emergence of reproductive isolation and speciation of *D*. *melanogaster* during evolution (Fig 1B). Indeed, only *D*. *melanogaster* has *Su(Ste)* and *AT-chX* repeats [7,21]. When *D*. *melanogaster* females mate with other *Drosophila* species males, *Stellate* genes cannot be repressed, and the hybrids flies are sterile. Intriguingly, *AT-chX* piRNAs have 76% homology to *vasa* transcripts within *D*. *melanogaster* but do not have the ability to repress vasa in the testis or ovaries. However, *vasa* sequences from related species that diverged from a *Drosophila* common ancestor several million years ago have more than 90% complementarity with piRNAs from *AT-chX* repeats of *D*. *melanogaster*. *AT-chX* piRNAs are more similar to “alien vasa” than endogenous vasa, suggesting that those piRNAs could help to preserve reproductive isolation and speciation through the repression of “alien vasa” when crossed to other species, since vasa is crucial for the piRNA pathway, germline formation, and fertility.

Thanks to their discovery, Chen and colleagues open the possibility to reconsider many other proteins of the piRNA pathway thought to be futile in males, which will help to better understand the global piRNA mechanisms and piRNA sexual dimorphism.
